# The Impact of Drop Test Conditions on Brain Strain Location and Severity: A Novel Approach Using a Deep Learning Model

**DOI:** 10.1007/s10439-024-03525-w

**Published:** 2024-05-13

**Authors:** George Stilwell, Danyon Stitt, Keith Alexander, Nick Draper, Natalia Kabaliuk

**Affiliations:** 1 Department of Mechanical Engineering, University of Canterbury, Christchurch, 8041 New Zealand; 2 Faculty of Health, University of Canterbury, Christchurch, 8041 New Zealand

**Keywords:** Concussion, Brain strain, Rugby, Rotational motion, Finite element

## Abstract

In contact sports such as rugby, players are at risk of sustaining traumatic brain injuries (TBI) due to high-intensity head impacts that generate high linear and rotational accelerations of the head. Previous studies have established a clear link between high-intensity head impacts and brain strains that result in concussions. This study presents a novel approach to investigating the effect of a range of laboratory controlled drop test parameters on regional peak and mean maximum principal strain (MPS) predictions within the brain using a trained convolutional neural network (CNN). The CNN is publicly available at https://github.com/Jilab-biomechanics/CNN-brain-strains. The results of this study corroborate previous findings that impacts to the side of the head result in significantly higher regional MPS than forehead impacts. Forehead impacts tend to result in the lowest region-averaged MPS values for impacts where the surface angle was at 0° and 45°, while side impacts tend to result in higher regional peak and mean MPS. The absence of a neck in drop tests resulted in lower regional peak and mean MPS values. The results indicated that the relationship between drop test parameters and resulting regional peak and mean MPS predictions is complex. The study’s findings offer valuable insights into how deep learning models can be used to provide more detailed insights into how drop test conditions impact regional MPS. The novel approach used in this paper to predict brain strains can be applied in the development of better methods to reduce the brain strain resulting from head accelerations such as protective sports headgear.

## Introduction

Traumatic brain injuries (TBI) are a significant public health issue, resulting from a bump, blow, or jolt to the head or a penetrating head injury. In the United States alone, TBI causes approximately 2.2 million emergency department visits annually [1, 2]. Several studies have recognised the connection between high-intensity head impacts and concussions [3–6]. Rugby players, for example, are exposed to an average of 14–52 head impacts per game, with a peak linear acceleration above 10 g [7–10]. Unsurprisingly, mild traumatic brain injuries (mTBI), commonly labelled as concussions, are one of the most frequent injuries sustained by rugby players [11–13]. Prolonged exposure to high-intensity head acceleration events have been linked to a number of long-term mood and cognitive deficits including neurodegenerative diseases such as chronic traumatic encephalopathy and early onset Alzheimer’s [14–20]. The reported incidence rates of concussion vary dramatically due to the ill-defined nature of what constitutes a “concussion” [21], with under-reporting rates of concussion estimated to be as high as 50-90% in contact sports such as rugby league [22, 23]. As a result, sport-related concussions have been described as a “silent epidemic” [24].

Attempts to quantify the risk of a given head impact resulting in an mTBI have led to the development of several brain injury criteria (BIC) [4–6, 25, 26]. The head injury criterion (HIC) and rotational injury criterion (RIC) are well-known examples of metrics that have been developed using time series kinematic measures of the head during the impact. The use of single variable metrics such as peak linear acceleration (PLA) and BIC alone, however, does not provide a detailed region-specific mechanical response of the brain [27]. Zhan et al. have shown the accuracy of a given BIC metric decreases when applied to a head impact context outside that in which it was developed [15]. Typically, BIC measures are too oversimplified to provide adequate detail of tissue-level insights of TBIs [28]. As a result, there is no consensus between researchers on an appropriate kinematics-based injury metric or a tolerance threshold for sports-related concussion [29]. It should be noted, however, that rotational motion has long been implicated as a primary driver of mTBI symptoms [30–32].

Many studies have shown brain strain, particularly maximum principal strain (MPS), to be a primary mechanism and thus an effective predictor of TBI [25, 31, 33, 34]. The development of dozens of finite element models have enabled researchers a more detailed method to analyse the mechanical response of the brain during an impact [35–38]. One study investigated the effect of impact location on the concussion risk of American football players using two FE models to evaluate the magnitude and distribution of regional brain strains [39]. Laboratory impact tests were used to gather kinematic data using two impact speeds and 12 impact directions. Results from the Simulated Injury Monitor (SIMon) and Global Human Body Model Consortium (GHBMC) models suggested that frontal impacts to the crown and forehead resulted in the lowest brain strain values [39]. Similarly, a study completed using kinematic data from head impact youth football players found that impacts to the top of the helmet were associated with lower strain metrics [40]. Impacts to the side of the helmet were associated with the highest peak rotational velocity and strain metrics [40]. These results corroborate with a study that compared the brain response of frontal and lateral impacts using FE modelling, with predicted shear stress in the brain being much higher in lateral impacts [41].

Due to the complexity of these models, simulations are resource intensive, with some requiring hours of computation time [42–44]. As a consequence, this has limited the routine use of models in practical applications, such as immediate TBI assessment of sports players following an on-field head impact [45]. Until recently, kinematic data from on-field impacts were only analysed days or weeks after the event [46]. Methods to reduce computation time often simultaneously reduce the accuracy of the model outputs, especially for large strain impacts [33, 47]. In contrast, the pre-computation technique put forward by Ji et al. has enabled element-wise, whole-brain MPS to be computed instantly [27]. Once trained on a large library of head impacts, machine learning head models (MLHMs) have enabled whole-brain MPS to be computed in seconds [48, 49]. Recently, a convolutional neural network (CNN) has been developed and trained using simulation results of head impacts using the Worcester Head Injury Model (WHIM) [50, 51]. The pre-trained CNN enables the nonlinear impact strain relationship to be computed in milliseconds based on the rotational velocity profile of the head [52]. Following the development of these initial MLHMs, a range of research groups have developed subsequent models for a range of applications such as the evaluation of protective helmets and the simulation of head impacts in situations including sports and traffic accidents [53–55]. Such models are capable of acting as a rapid estimation FE model and could accelerate the understanding of mTBI during future studies. Although MLHMs are able to rapidly estimate brain stains, the accuracy of these types of models has been shown to be dependent on the situation in which they are applied in relation to the head impact training data that were used to develop the model [56, 57]. Model accuracy has been shown to decrease when the training datasets are from a range of different impact types such as car accidents, boxing, and college football [56, 57]. Therefore, care needs to be taken when interpreting the results from these models, especially when the head impact training data of the model differ from the application when the model is being employed.

Previous work investigating the effect of drop test condition on the shape of the kinematic profiles of a Hybrid III (HIII) headform found rotational velocity to be largely unaffected [58]. This study aimed to deepen this analysis by investigating the relationship between drop test condition on the location and severity of MPS within the brain. The results of this study could be applied to better understand the relation between field and lab head impact conditions and may act to better understand the biomechanics behind injurious gameplay.

## Materials and Methods

### Experimental Procedure

**Fig. 1:**
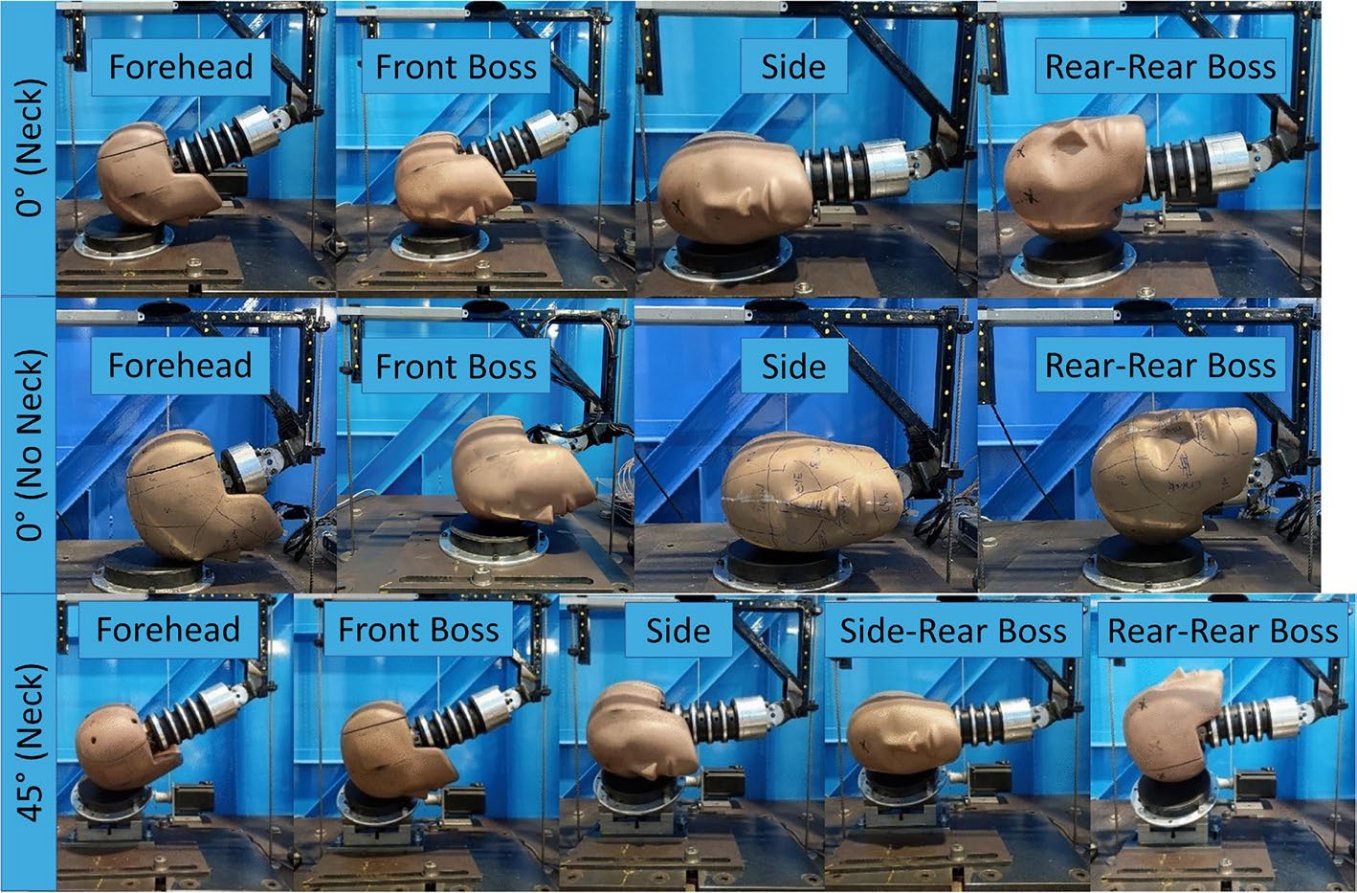
Drop test conditions for the 0° and 45° impact surfaces used in the study.

All impacts were carried out on a twin wire guided drop test rig using a HIII headform (50th percentile male model) instrumented with a nine accelerometer package (NAP) [59]. Three variations of the drop test method were carried out for comparison based on previous work by Stitt et al. and Draper et al. The first used a HIII head and a standard 1-inch Modular Elastomer Pad (MEP) for the impact surface (manufactured by Cadex Inc.), with no neck involved [58, 60]. The second and third drop test variations used were taken from the same authors’ study of rugby headgear [61]. Using the same HIII head and neck, and standard 1-inch MEP pad impact surface, drop tests were carried out with the impact surface angled at 0° and 45° relative to the test rig base. For all tests, the MEP pad was securely bolted to the base of the drop tower. The MEP pad did not slip or separate from the impact surface during laboratory testing. All three drop test variations were carried out across 4 impact locations: forehead (sagittal rotation), front boss, side (coronal rotation), and rear boss (labelled rear-rear boss), as shown in Figure 1. The front boss and rear boss impacts induce multiaxial rotations. Impacts onto the 45° impact surface also included a fifth impact location labelled side-rear boss. All impacts were completed in the orientations shown in Figure 1. With the exception of the rear-rear boss impact for the 45° impacts, all impacts were on the right side of the head. Care should be taken when comparing the resulting strain predictions to the left and right side of the cerebrum for this impact condition compared to the others used in the study. The combination of each impact location and impact surface angle will be hereon referred to as different impact *conditions*. Each impact condition was repeated 5 times at each drop height, with 60 seconds between each repeat.

Unlike the cited studies, trends between drop heights were assumed to be adequately captured by two drop heights. For this reason, only drop heights of 150 and 450 mm were used in this study. Impacts onto the 45° impact surface were assumed to create motions that were only allowable by a flexible neck. The occipital condyle joint on the HIII head does not permit such movement; therefore, 45° impacts without the neck were excluded from the study. A summary of the drop test conditions is outlined in Table 1. To determine the impact energy, the mass of the head and neck of 5.6 kg and the total falling mass of 6.8 kg (including the drop frame) were used.

### Data Acquisition and Post Processing

The HIII headform was instrumented with four triaxial accelerometers (Analog Devices ADXL377, 20*,*000 Hz, range: ± 200 g, sensitivity: 6*.*5 mV*/g*) creating a NAP with three redundant sensing axes. This allowed linear and rotational accelerations, and rotational velocity, to be measured and calculated [59]. Data from the accelerometers were recorded by a LabVIEW system and post-processed in Matlab and Python 3.8. All of the kinematic data that have been reported and used in this study were filtered using the default 8th-order Butterworth low-pass filter from the “scipy.signal” library of Python. A cutoff frequency of 300 Hz was used to reduce noise in the measured kinematics signals. The 300 Hz frequency was used as this frequency is commonly used by head impact researchers that use similar sensors [62, 63]. Peak kinematics were defined as the maximum value of the resultant trace of the kinematic as an average across the five impact condition repeats.

### Estimates of Regional Peak and Mean MPS using Convolutional Neural Network

Regional peak and mean MPS were predicted using the publicly available pre-trained convolutional neural network (CNN) which can be found at https://github.com/Jilab-biomechanics/CNN-brain-strains. The trained CNN developed by Wu et al. (2019) to estimate regional brain strains was selected over other MLHMs in this study as it is publicly available, provides regional brain strain estimations with sufficient accuracy, and was trained on two datasets that included impacts from American football which is a similar sport to rugby [52]. The training dataset also included lab-reconstructed impacts from National Football League which are similar to the drop tests that were used in this study. The CNN was trained on head impacts simulated with the WHIM finite element model of the head. Preprocessing of the head impact time series rotational velocity data was carried out using the preprocessing codes published in the repository, thus ensuring the azimuth and elevation of rotational velocity axes matched that required for the CNN model. The CNN only requires rotational kinematics to estimate the peak and mean MPS values in the brain. This simplification is supported by the finding that linear kinematics have a minimal effect on the peak and distribution of strain throughout the brain [64].

### Statistical Analysis

To assess whether the impact locations, impact angles, the inclusion of a neck, and drop height effect regional peak and mean MPS values, multiple two-way analysis of variance (ANOVA) tests were completed in R using the five data points from each impact condition. Peak and mean MPS values were analysed to determine the effect of impact location (4 levels), neck inclusion (2 levels), and impact angle (2 levels). Multiple pairwise-comparisons were competed using the post hoc Tukey’s Honest Significant Difference (HSD) tests when significance was found. The confidence interval was set to 95% (*p* < 0.05) for the statistical analysis. When comparing the 0° and 45° results, the side-rear boss result was not included to keep the analysis balanced.

## Results

### Head Kinematics

The kinematic measures of each drop test condition are shown in Table 1. The PLAs varied from 33.2 to 122.3 m/s^2^, peak rotational accelerations (PRAs) varied from 1700 to 12030 rad/s^2^, and peak rotational velocities (PRVs) from 10.4 to 32.9 rad/s. For clarity, the maximum values in each row group of Table 1 have been highlighted in bold. As expected, drop tests from a height of 450 mm resulted in larger peak kinematics. The largest peak rotational velocities for drop tests with an impact angle of 0° occurred during impacts to the side of the head. For drop tests with an impact angle of 45°, the largest PRVs occur as a result of a side-rear boss impact location, followed by impacts to the side.

**Table 1:** Impact kinematics; Mean (SD)

Drop heights mm	Impact angle	Impact location	PLA g	PRA rad/s^2^	PRV rad/s	Impact velocity m/s	Impact energy J
150	0°	Forehead	60.4 (0.2)	2790 (25)	14.3 (0.1)	**1.69 (0.007)**	**9.68 (0.003)**
		Front Boss	50.1 (0.3)	3350 (10)	13.4 (0.1)	1.65 (0.004)	9.31 (0.001)
		Rear-Rear Boss	53 (0.2)	3360 (16)	**15.4 (0.3)**	1.66 (0.01)	9.33 (0.004)
		Side	56.1 (0.4)	5330 (38)	17.7 (0.1)	1.64 (0.01)	9.15 (0.003)
		Forehead (No Neck)	49.9 (0.5)	2380 (89)	10.4 (0.3)	1.67 (0.01)	8.1 (0.004)
		Front Boss (No Neck)	43.2 (0.7)	3470 (129)	10.5 (0.8)	1.65 (0.01)	7.91 (0.004)
		Rear-Rear Boss (No Neck)	46.8 (0.5)	3780 (109)	12.1 (0.2)	1.68 (0.01)	8.19 (0.005)
		Side (No Neck)	**67.7 (9.8)**	**5600 (251)**	13 (1.3)	1.64 (0.02)	7.82 (0.007)
150	45°	Forehead	34.5 (1.1)	2660 (135)	12.8 (0.5)	1.66 (0.03)	9.36 (0.01)
		Front Boss	33.2 (0.6)	1700 (30)	13 (0.1)	1.66 (0.02)	9.41 (0.006)
		Rear-Rear Boss	**41.3 (0.2)**	2040 (27)	15.4 (0.09)	**1.73 (0.009)**	**10.13 (0.003)**
		Side	33.5 (1.3)	**4240 (327)**	17.4 (0.8)	1.69 (0.01)	9.71 (0.004)
		Side-Rear Boss	29.7 (1.0)	4030 (350)	**19.1 (1.0)**	1.67 (0.02)	9.52 (0.007)
450	0°	Forehead	119.6 (0.7)	4900 (90)	28.5 (0.2)	2.89 (0.02)	**28.3 (0.003)**
		Front Boss	112.3 (1.3)	7830 (276)	25.4 (0.1)	2.86 (0.01)	27.89 (0.002)
		Rear-Rear Boss	115.6 (0.3)	8360 (165)	28.4 (0.3)	2.81 (0.01)	26.84 (0.003)
		Side	**123.4 (1.9)**	**12030 (152)**	**32.9 (0.4)**	2.75 (0.02)	25.74 (0.004)
		Forehead (No Neck)	122.1 (11.0)	5290 (455)	21.3 (1.5)	**2.93 (0.03)**	25.34 (0.006)
		Front Boss (No Neck)	102.6 (1.9)	7640 (445)	21.4 (1.8)	2.88 (0.06)	24.49 (0.02)
		Rear-Rear Boss (No Neck)	104.5 (2.5)	8500 (736)	23.3 (1.3)	2.92 (0.004)	25.13 (0.001)
		Side (No Neck)	110.4 (13.8)	10670 (1644)	30.7 (0.9)	2.4 (0.9)	16.96 (0.2)
450	45°	Forehead	**84.5 (0.3)**	6230 (60)	23.4 (0.14)	2.77 (0.02)	26.13 (0.003)
		Front Boss	79.4 (1.4)	3050 (87)	20.5 (0.6)	**2.89 (0.02)**	**28.31 (0.005)**
		Rear-Rear Boss	79.8 (0.6)	3830 (174)	25.2 (0.2)	2.65 (0.02)	23.82 (0.005)
		Side	72.5 (0.3)	8260 (155)	27.8 (0.4)	2.78 (0.02)	26.37 (0.004)
		Side-Rear Boss	61.8 (0.2)	**8460 (137)**	**31.2 (0.1)**	2.61 (0.02)	23.17 (0.005)

### Effect of Drop Test Condition on Strain Distribution

Volume fractions of the rapid estimation FE model within specified MPS ranges are shown in Table 2 for each drop test condition. A large difference was observed in the distribution of MPS between the two drop heights. As expected, condition-matched impacts from 450 mm resulted in a higher proportion of the brain experiencing more severe levels of MPS than those from 150 mm. Notably, volume fractions of the brain greater than 0.1% experiencing MPS greater than 30% were not observed for 150 mm drop tests. At both drop heights, however, impacts to the side of the head resulted in the largest distribution of high MPS, followed by rear-rear boss, front boss, and finally, forehead, for impacts onto the 0° impact surface (both with and without a neck).

**Table 2:** Mean (SD) volume fractions of the brain within different strain ranges

Drop heights mm	Impact angle	Impact loction	Volume fraction of brain between specified strain ranges %				
			0–10	10–20	20–30	30–40	40–50
150	0°	Forehead	88.8 (0.11)	11.2 (0.11)	–	–	–
		Front Boss	77.7 (0.71)	22.2 (0.7)	–	–	–
		Rear-Rear Boss	70.5 (0.49)	29.5 (0.49)	0.1 (0.01)	–	–
		Side	67.3 (0.49)	32.6 (0.48)	0.1 (0.01)	–	–
		Forehead (No Neck)	95.1 (0.25)	4.9 (0.25)	–	–	–
		Front Boss (No Neck)	91.7 (2.44)	8.3 (2.44)	–	–	–
		Rear-Rear Boss (No Neck)	80.3 (3.87)	19.7 (3.86)	–	–	–
		Side (No Neck)	71.8 (6.01)	28.1 (5.96)	0.1 (0.04)	–	–
150	45°	Forehead	68.6 (3.68)	31.2 (3.48)	0.2 (0.22)	–	–
		Front Boss	79.1 (5.73)	20.5 (5.44)	0.4 (0.29)	–	–
		Rear-Rear Boss	81.6 (0.73)	18.3 (0.73)	0 (0.01)	–	–
		Side	51.1 (3.02)	45.7 (2.48)	3.2 (0.59)	–	–
		Side-Rear Boss	40.6 (2.8)	54.8 (1.65)	4.5 (1.16)	–	–
450	0°	Forehead	32.9 (0.75)	59.4 (0.5)	7.5 (0.25)	0.2 (0.03)	–
		Front Boss	23.1 (0.18)	58.1 (0.87)	17.5 (0.86)	1.3 (0.12)	–
		Rear-Rear Boss	14.7 (1.3)	65.2 (0.3)	18.1 (0.86)	2 (0.25)	–
		Side	6.3 (0.33)	64.3 (0.33)	25.1 (0.33)	4.1 (0.19)	0.2 (0)
		Forehead (No Neck)	30.1 (9.34)	63.4 (6.85)	6.2 (2.45)	0.2 (0.17)	–
		Front Boss (No Neck)	20.5 (6.39)	70.9 (4.77)	8.5 (2)	0.1 (0.01)	–
		Rear-Rear Boss (No Neck)	11.6 (2.73)	74.6 (0.88)	13.4 (1.68)	0.4 (0.2)	–
		Side (No Neck)	5.5 (2.3)	68.1 (5.59)	23.6 (6.55)	2.6 (1.28)	0.1 (0.04)
450	45°	Forehead	20.4 (0.61)	58 (0.37)	19.1 (0.36)	2.4 (0.07)	0 (0.01)
		Front Boss	51.4 (4.08)	46.3 (3.25)	2.3 (0.76)	–	–
		Rear-Rear Boss	22.1 (0.49)	62.1 (0.28)	14 (0.29)	1.8 (0.12)	–
		Side	9.4 (0.27)	52 (0.59)	32.6 (0.43)	5.7 (0.07)	0.3 (0.01)
		Side-Rear Boss	16.6 (0.59)	64.9 (0.93)	16.2 (1.03)	2.4 (0.46)	–

However, this trend was not observed for the 45° tests, as the front boss impact resulted in the largest volume fraction of strain within the 0-10% range for both the 150 mm and 450 mm drop tests. The side impact was the most severe impact location, having the largest volume fraction of strain within the 30-40% range for the 450 mm drop tests and a similar result in the 10-20% range for the 150 mm drop tests.

**Fig. 2:**
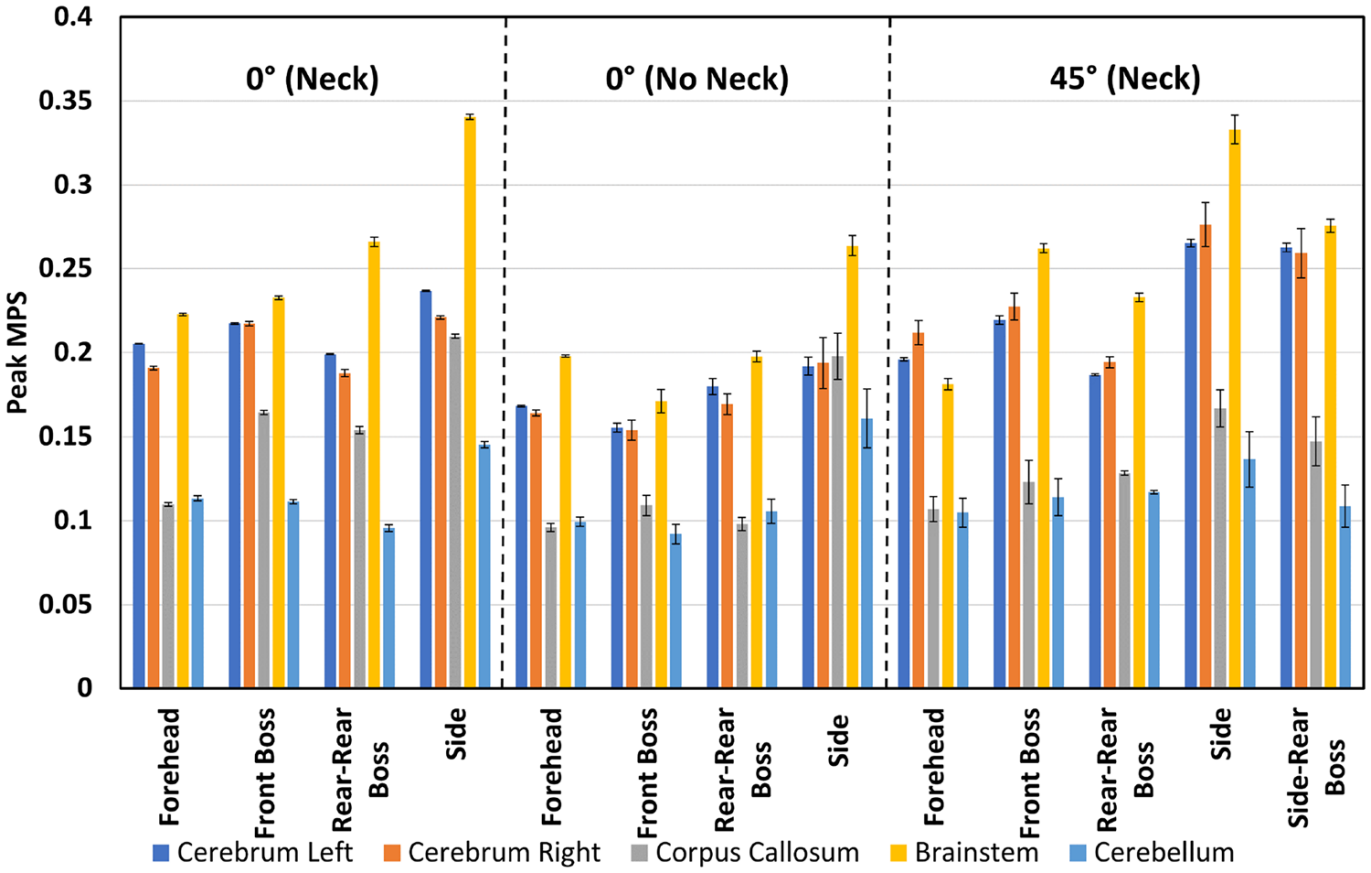
Peak strain for each drop test condition from 150 mm.

**Fig. 3:**
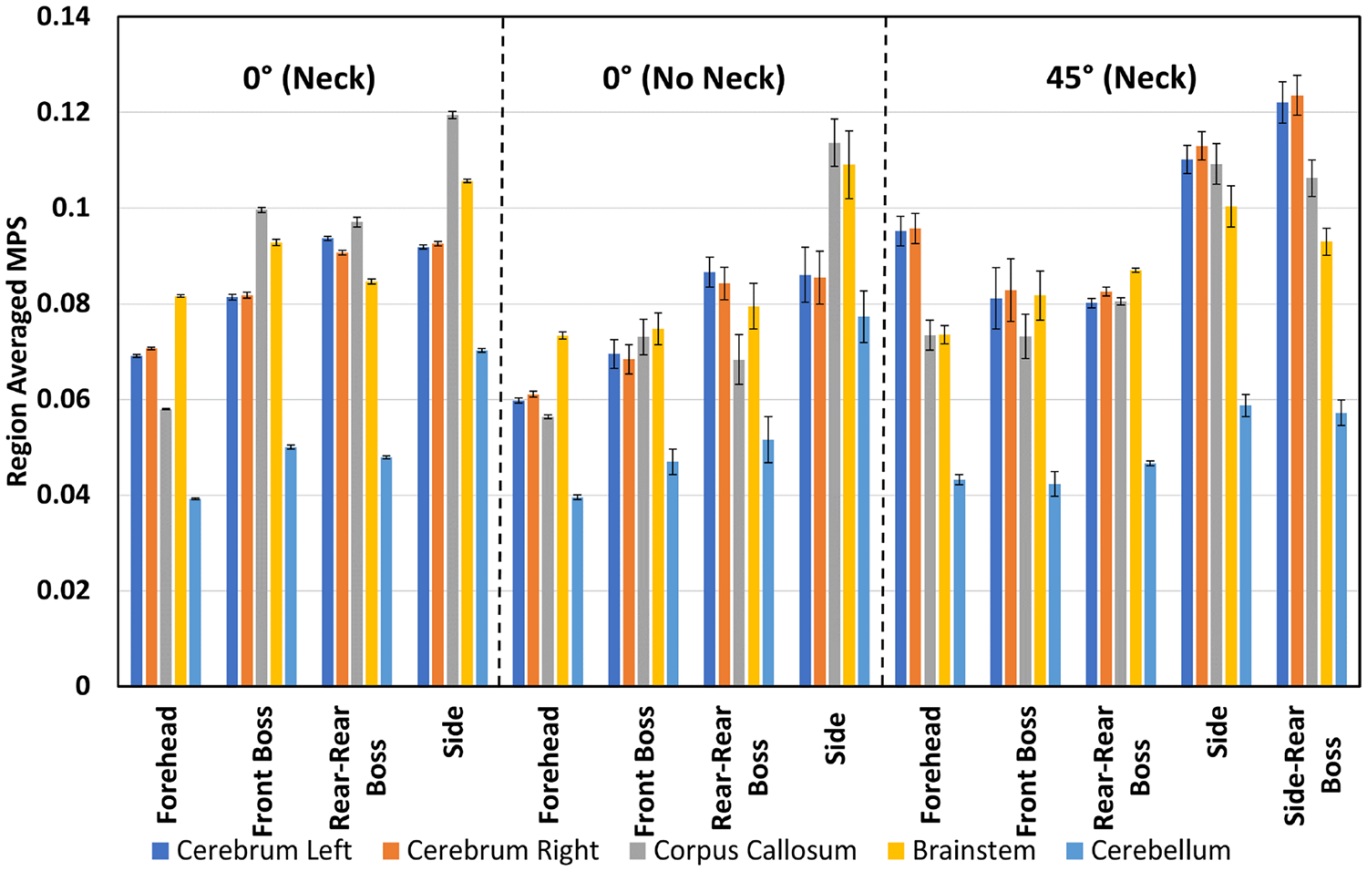
Region-averaged MPS for each drop test condition from 150 mm.

**Fig. 4:**
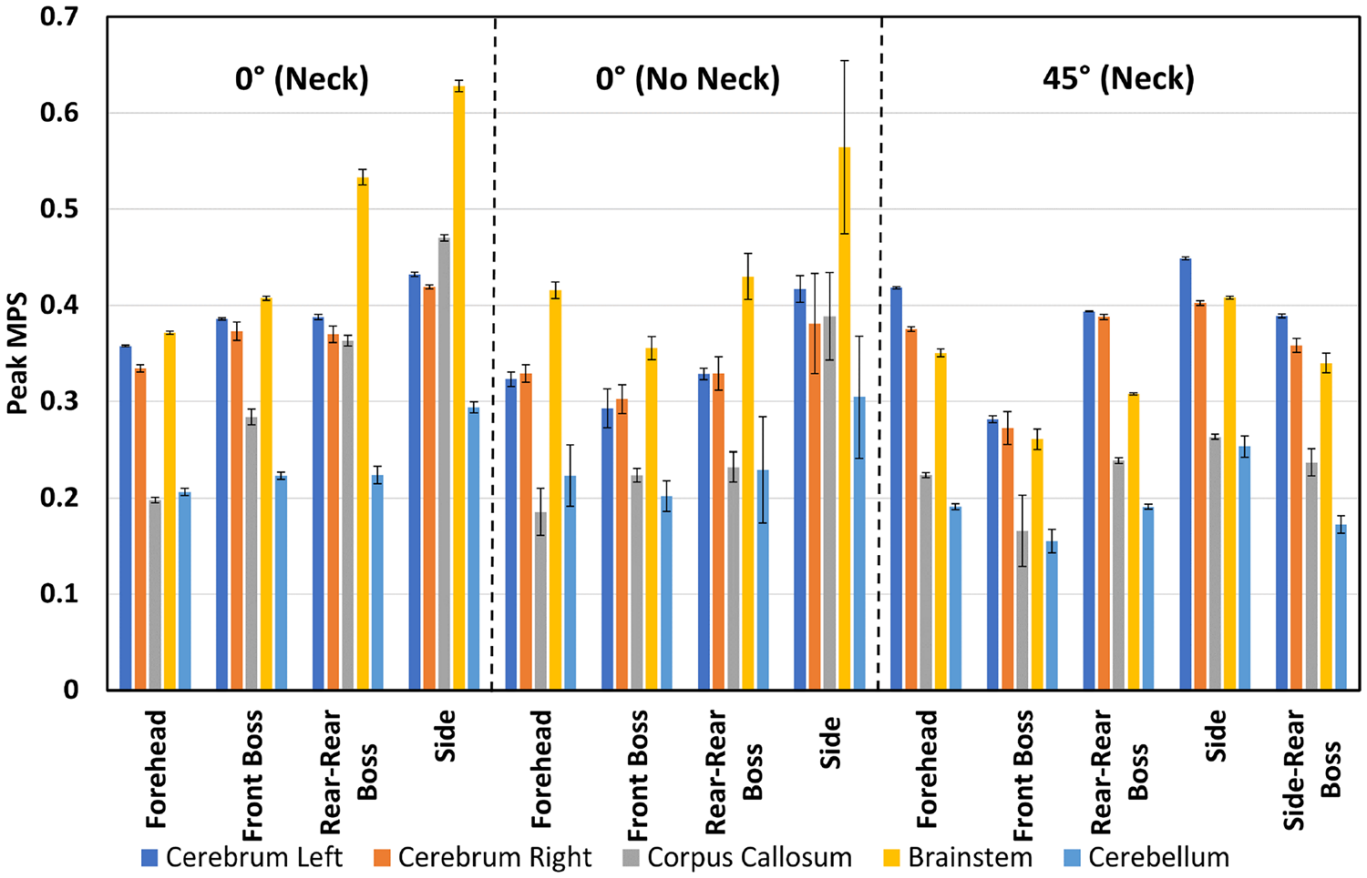
Peak strain for each drop test condition from 450 mm.

**Fig. 5:**
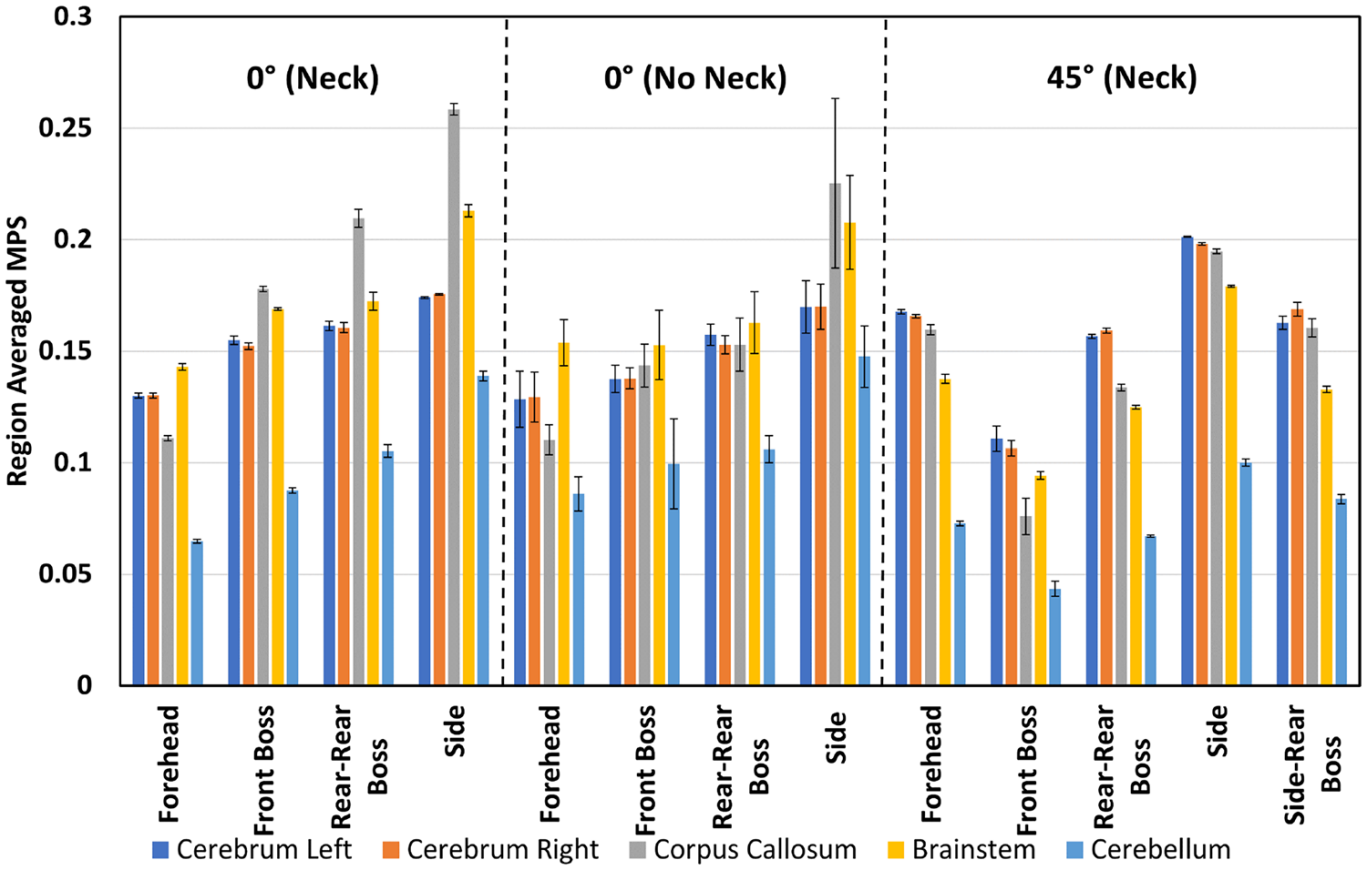
Region-averaged MPS for each drop test condition from 450 mm.

Figures 2, 3, 4, and 5 present the regional peak and region-averaged MPS for each drop condition and height grouped by test condition (angle and inclusion of neck). These Figures show the maximum MPS values tended to occur in the brainstem for both drop heights, with 92% of the 150 mm impacts and 62% of the 450 mm impacts resulting in maximum peak strain values in this area. In contrast, the cerebellum had the lowest peak MPS values, with 77% of all drop tests resulting in lower peak strains in this region. The peak MPS values ranged from 0.16 to 0.63 for the 450 mm drop tests and 0.09 to 0.34 for the 150 mm drop tests across all drop tests.

Figures 2 and 4 show large differences in regional peak MPS within each drop test condition. Specifically, impacts to the side of the head resulted in the highest peak MPS values for the 0° neck, no neck, and 45° 150 mm drop conditions, with peak MPS of 0.34, 0.26, and 0.33, respectively, all occurring in the brainstem. When interpreted with the volume fraction data in Table 2, it was observed that peak strain occurred in less than 0.1% of the total brain volume. Similarly, the 0° neck and no neck 450 mm drop tests resulted in the highest peak strain values of 0.63 and 0.56, respectively, also occurring in the brainstem. The 45° 450 mm drop test condition resulted in a peak strain of 0.45, which occurred in the left side of the cerebrum from an impact to the side of the head.

Figures 3 and 5 show the region-averaged values of MPS in each brain region. Although the peak value of MPS is of most interest, the region-averaged MPS in each region offers an indication as to the distribution of MPS values throughout each region. Across 83% of the drop test conditions, the largest region-averaged MPS values occurred as the result of an impact to the side of the head. Region-averaged MPS values varied from 0.04 to 0.26 for the 450 mm drop tests and 0.04 to 0.12 for the 150 mm drop tests.

### Effect of Impact Location

Analysis of the 150 mm results revealed that impact location had a significant effect (*p* < 0.05) on the region-averaged and peak MPS values for each region of the brain. However, in almost all of the two-way ANOVA tests that compared the impact location with the impact angle and neck inclusion for the 150 mm drop tests, interaction effects were present. The presence of interaction effects indicates that the relationship between drop test parameters and the resulting MPS metrics is complex.

The analysis of the 150 mm drop tests comparing the effect of impact location and neck inclusion on the region-averaged MPS in the left cerebrum did not show any interaction effect. Impacts to the forehead resulted in the smallest region-averaged MPS in the left side of the cerebrum, followed by front boss impacts. In contrast, impacts to the side of the head and rear-rear boss locations resulted in the largest region-averaged MPS values for both the inclusion and exclusion of the neck. Furthermore, the p-value of 0.79 obtained from the Tukey’s test between the side and rear-rear boss impact location indicated that there was no significant difference in region-averaged MPS in the left side of the cerebrum caused by these two impact locations.

Analysis of the results of impact location and surface angle for the 150 mm drop height all showed the presence of interaction effects. The interaction effects for the region-averaged MPS were often complex and disordinal due to the variations in trends for different areas of the brain between the impacts where the impact surface was angled at 0° and 45°. The differences between the region-averaged values for drop tests where the impact surface was angled at 0° and 45° were small and ranged from 0 to 0.03. The results showed that forehead impacts resulted in the lowest region-averaged MPS values for impacts where the surface was angled at 0° and 45°, while side impacts resulted in the highest region-averaged MPS values. For the 45° impacts, side impacts resulted in the highest region-averaged MPS values. However, the trends for the other impact locations were inconsistent across different brain regions.

The trends for peak strain values varied across different brain regions. In the left and right cerebrum, the difference between peak strains caused by forehead and front boss was similar, ranging from 0.19 to 0.23. However, side impacts resulted in the highest peak strain values, with 45° impacts leading to significantly higher strain values in the left and right cerebrum (0.27 and 0.28) than 0° impacts (0.24 and 0.22). In contrast, 45° impacts resulted in significantly lower peak strain values in the corpus callosum for front boss, rear-rear boss, and side impacts. No significant difference was observed in peak strain values for 45° and 0° forehead impacts.

The location of impact had a significant effect on the region-averaged and peak MPS values for all 450 mm impacts. Forehead impacts consistently resulted in smaller region-averaged MPS values ranging from 0.065 to 0.15, with front boss and forehead impacts producing the smallest peak strain values. In contrast, side impacts consistently resulted in larger region-averaged and peak MPS values, with peak MPS reaching 0.63 in the brainstem. Results from drop tests where the neck was included typically produced equal or larger peak strain values than identical drop tests that excluded the neck.

The 450 mm drop test results revealed that impact angle and location both have a significant effect on the region-averaged and peak MPS values for all impacts. Similar to the results from the 150 mm drop test, interaction effects were present in the results. The 0° impacts tended to follow the trend of forehead impacts resulting in the lowest region-averaged and peak MPS values, while side impacts resulted in the largest values. Interestingly, for 45° impacts, the front boss impact resulted in the lowest region-averaged and peak MPS values. Across all brain regions, the peak strain values for 450 mm drop tests varied from 0.19 to 0.28. Side impacts consistently produced the highest region-averaged and peak MPS values compared to other impact locations, with peak strain ranging from 0.25 to 0.45 for 45° side impacts.

### Effect of Surface Angle

The results of the 150 mm drop tests indicate that surface angle had a significant effect on region-averaged MPS values in all regions of the brain, while only showing significant differences in peak strain values in the right side of the cerebrum, corpus callosum, and brainstem. Tukey’s tests demonstrated that the 45° impact surface resulted in higher region-averaged MPS values in the left and right side of the cerebrum, and lower region-averaged MPS values in the corpus callosum, brainstem, and cerebellum. Additionally, the 45° impact surface led to larger peak strain values in the right side of the cerebrum and lower peak strain values in the corpus callosum and brainstem.

Similarly, the 450 mm drop tests also demonstrated significant effects of surface angle on region-averaged and peak MPS values in all regions of the brain except for the left side of the cerebrum. The results of Tukey’s tests for the 450 mm drop tests follow the same trends as the 150 mm drop tests, with the 45° impact surface leading to higher region-averaged MPS values in the left and right side of the cerebrum, and lower region-averaged MPS values in the cerebrum, corpus callosum, and brainstem. Moreover, the peak strain values were smaller when a 45° impact surface was used. These findings suggest that surface angle should be taken into account when designing and evaluating protective measures for brain injury.

### Effect of Neck Inclusion

Analysis of the 150 mm results revealed that the inclusion of the neck had a significant effect on the region-averaged and peak MPS values for all areas of the brain except for the peak strain in the cerebellum. The results from the post hoc Tukey’s test show that the inclusion of the neck results in higher region-averaged MPS values for all brain regions except for the cerebellum. The trends for the peak strain values follow the same trends, with all regions having higher peak strain values with the inclusion of the neck except for the peak strain in the cerebellum where there was no significant difference between the inclusion and exclusion of the neck.

Analysis of the 450 mm results showed that the inclusion of the neck had a significant effect on the region-averaged and peak MPS for all impacts except the region-averaged MPS in the brainstem and peak strain in the cerebellum. In particular, the inclusion of the neck resulted in lower region-averaged MPS values for the cerebellum but higher region-averaged and peak MPS values for all other regions. The largest difference in peak strain values occurred in the corpus callosum, where a rear-rear boss impact resulted in a peak strain of 0.23 when the neck was not included, and 0.36 when the neck was included. The differences between the neck and no-neck peak strains were larger than the region-averaged MPS values.

## Discussion

The focus of this study was to investigate the effects that drop test parameters have on regional peak and mean MPS location and severity. To complete this, five impact locations were tested at two impact angles, two drop heights and both including and excluding the neck. To generate the results in this study, the velocity profiles obtained from the controlled lab impacts were input into a pre-trained CNN model [52] to rapidly estimate regional peak and mean MPS values. The resulting regional peak and mean MPS values found that the drop test parameters of the impact location, impact angle, and the inclusion of the neck all had a significant result on the mean and peak regional MPS values. However, interaction effects were often present when analysing the pairs of drop test parameters, indicating that the relationship between drop test parameters and the resulting regional peak and mean MPS is complex.

### Effect of Drop Height

When looking at the effect of increasing drop height on strain values across all regions of the brain, the results showed that an increase in drop height led to significantly higher impact energy as shown Table 1. As the impact energy increases, the head experiences larger forces and accelerations, leading to greater deformation of brain tissue and higher strain values. This relationship between impact energy and brain strain has been documented previously [39]. However, when excluding side-rear boss impacts, impacts to the side of the head consistently resulted in the larger regional peak and mean MPS values. These results show that impact energy alone does not directly determine the resulting regional peak and mean MPS, thus indicating a more complex interaction between variables. This highlights that factors such as impact location and impact compliance (the overall stiffness of an impact) play a critical role in the resulting head kinematics [39, 58, 61] and thus brain injury. Interestingly, the trends between impact location and resulting strain were not always consistent between the 150 mm and 450 mm drop heights used in this study.

### Effect of Impact Surface Angle

When considering the head kinematics in Table 1, the results showed that the 450 mm drop tests resulted in the highest peak kinematics. Impact location and angle significantly influenced peak rotational velocity, with the largest peak rotational velocity of 32.9 rad/s for drop tests with an impact angle of 0° occurring as a result of an impact to the side of the head, and for drop tests with an impact angle of 45°, the peak rotational velocities of 31.2 rad/s occurred as a result of a side-rear boss impact location.

The difference in rotational velocities and regional peak and mean MPS from side-rear boss and side impacts compared to frontal impacts can be attributed to the oblique nature of the force vector during the collision. As the impact force is not applied in line with the centre of mass of the brain, a rotational moment is applied to the brain. This rotational moment induces internal shear forces and shear-induced tissue damage [14]. There is considerable literature that discusses the rotational kinematics of the head as a main mechanism for TBI [65–68]. The results from the drop tests in this paper corroborate with these studies in terms of predicted MPS. The results of this paper also highlight the complex interactions between each of the laboratory drop test parameters.

### Effect of Neck Inclusion

Overall, the inclusion of the neck in the drop test results had a significant effect on the region-averaged and peak MPS values for all areas of the brain except for the peak strain in the cerebellum. In both the 150 mm and 450 mm drop tests, the inclusion of the neck resulted in higher region-averaged and peak MPS values for most regions of the brain. However, the inclusion of the neck resulted in lower region-averaged MPS values for the cerebellum in the 450 mm drop tests.

The additional mass of the neck is unlikely to be the reason for higher strain levels when the neck is included. As mass is added to the impacting body, the peak kinematics generally decrease. The inclusion of the neck extended the duration of the rotational velocity peak (i.e. the duration and shape of the rotational acceleration was affected, but not the peak). No measurable effect on the linear acceleration was observed. This result conforms with previous work that has investigated how neck inclusion impacts the kinematics of the headform. Sitt et al. [58] have shown that inclusion of the neck during drop testing did not significantly influence the peak linear and rotational accelerations, or the peak rotational velocity for both height- and energy-matched impacts. It did, however, significantly influence the shape of the rotational velocity kinematic trace and, by extension, the rotational acceleration trace. Inclusion of the neck during drop testing extended the duration of the rotational velocity, especially in the side impact location, where the occipital condyle joint of the HIII head restricts rotational motion in the direction of the impact force. This extension of the duration of the rotational velocity peak likely explains the higher MPS values seen between impact with and without the neck. In addition, muscle activation of the neck has been shown to reduce the severity of TBI [69]. Such factors could provide a basis future work that could look to incorporate when investigating brain strains and TBI in rugby.

### Comparisons with Previous Studies

There are several studies that have investigated peak MPS values using simulations based on measured head kinematics [39, 70, 71]. Typical peak MPS values from these studies have ranged from 0.1 to 0.55. The results from this study are within a similar range, with peak strain values ranging from 0.09 to 0.63. The results from this study showed that impact location significantly altered the resulting kinematics and, by extension, peak and mean regional MPS values. In agreement with previous literature [39–41], impacts to the side of the head resulted in significantly higher regional MPS compared to forehead impacts.

### Limitations and Future Work

The current study presents a novel approach to investigating the effects of various drop test parameters on regional peak and mean MPS using lab-controlled impacts. However, several limitations should be noted. Firstly, the accuracy of the CNN MLHM to make regional MPS predictions is one limitation of the study. As the CNN model was trained on some datasets that included boxing, and mixed martial arts impacts, this is source of inaccuracy for the resulting MPS predictions. The CNN was used as it provides novel insights into what the resulting brain strain distribution may look like for the different drop test conditions. This CNN MLHM was used as it provides far greater insights than head impact kinematics or head injury criteria alone. However, care should be taken when trying to extrapolate the results from this model to actual brain injuries in sport, as these are unlikely to be exactly the same as what would develop in the brain. The focus of this study was not to see a human head responds, instead the focus was to investigate differences in regional MPS predictions with a range of laboratory drop test conditions. The MPS prediction is an additional metric that provides a close insight to injury. Secondly, the use of the HIII headform results in some limitations, as different headform shapes and sizes have been found to result in significantly different PRAs and PRVs [72, 73]. As the HIII headform is likely to have a different response to a real human head, it is difficult to correlate the drop test responses to that of a human head. As the CNN model uses the rotational velocity profile as an input, the use of a headform will result in different strain predictions to that of a human head. Although these predictions might not be exactly the same as what we would expect in a human head, the trends and regional strain predictions provide insight into what we might expect to see. The headform used in this study does not account for individual differences in brain tissue properties, which may also affect the accuracy of the results. Previous work has shown that brain size has a significant impact on injury susceptibility, with larger brains tending to have a higher risk of injury [74]. However, the relationship between brain size and injury risk is not always strictly linear, as other factors can also play a role. Thirdly, the study only investigated a limited range of drop test parameters including impact location and angle, drop height, and the inclusion of a neck. Other important factors, such as differences in loading rate due to the compliance of the impact surfaces, were not investigated. Weight differences were also not included as a parameter for this study, a study by Gimble and Hoshizaki found that the PLA decreased when head mass was increased for the same impact velocity [75]. Lastly, it is worth noting that the use of lab-controlled drop tests may not fully reflect the complexity and variability of real-world head impacts. While lab-controlled experiments allow for the systematic investigation of different parameters, they may not fully capture the randomness and variability of on-field impacts. By considering the impact conditions and injury mechanisms specific to TBIs in rugby, future work could investigate a wider range of impact parameters to identify the specific variables that cause increases in predicted brain strains. Knowledge of these variables will be used to guide helmet innovations and improve the quality of helmet protection to reduce the resulting brain strains incurred from playing rugby. The approach of using a CNN to predict regional peak and mean MPS brain strain values provides an additional metric for developing protective equipment such as headgear in rugby. The brain strain values also enable comparisons between different injury prevention methods, such as helmets, to be evaluated in terms of brain strain rather than other brain injury criteria which do not give a region-specific assessment of injury.
